# Inhalation of Ether

**Published:** 1847-07-01

**Authors:** 


I. Inhalation of Ether. By *J. Mason Warren*, M.D., one of the Surgeons of the Massachusetts General Hospital. Octavo, pp. 18.

II. Practical Remarks on the Inhalation of the Vapour of Sulphuric Ether. By *W. Philpotts Brookes*, M.D., Surgeon to the Cheltenham General Hospital, &c. &c. Octavo, pp. 68. Churchill, 1847.

It is a pleasing duty to have to record that the general result of the immense number of experiments upon the effects of the Inhalation of Ether, which have been repeated throughout entire Europe during the last few months, has been of the most satisfactory character—that expectations have not been disappointed, or fears and forebodings realized. We are not however aware that there are any new facts, whether as regards the *modus operandi* of the substance or the description of cases to which its employment may be extended, that call for notice. For some time to come the cautious observation and candid record of the various phenomena that present themselves will constitute the most important and responsible duty of those whose opportunities are numerous; for, at present, we are but on the threshold of the investigation. Amid the points towards which attention may be yet advantageously directed is, the conferring greater simplicity and portability upon the apparatus employed; for innumerable as are the varieties of those hitherto invented, all we have seen are defective in these essentials. We observe M. Roux of Toulon (*Gazette des Hôpitaux*, No. 61) speaks highly of a bag which he employs, shaped like a lady's reticule, and lined with a pig's bladder, which he says is very easily breathed from even by young children. M. Sichel (who discountenances ether in the more delicate operations of the eye), in some cases in which he desired to subdue excessive spasm of the eyelids preventive of the extraction of foreign bodies, and having no apparatus at hand, poured the ether into a teapot, and directed the patient to inspire through the spout, with success.

The two pamphlets before us contain, besides the detail of some cases, no observations with which the profession is not already well acquainted. That of Dr. Brookes is not at all to our taste. He says, in his preface, "I have never, in any way, endeavoured to shroud this question with mystery; for I have freely and willingly invited any professional or non-professional gentleman to view the operations that have fallen under my notice. These few pages have, therefore, been written as much for the general as for the medical reader."

It is little to Dr. Brookes' credit to have converted the operating theatre into a show-box for all the idlers of Cheltenham. We are as little willing to advocate mystery in this ether question as can be; but we are still less disposed to countenance its being made a vehicle for puffery. His pamphlet has not been written "*as much* for the general reader," but *entirely* for him.

